# Preliminary pharmacokinetic and psychophysical investigations after controlled oral and inhalative consumption of hexahydrocannabinol (HHC)

**DOI:** 10.1038/s41598-025-93931-4

**Published:** 2025-03-24

**Authors:** Lisa Höfert, Benjamin Franz, Cedric Groß, Delen Kuntze, Bronislav Jurásek, Martin Kuchař, Jan Dreßler, Susen Becker, Sven Baumann

**Affiliations:** 1 Institute of Forensic Medicine, Medical Faculty, University of Leipzig, Leipzig, Germany; 2 Forensic Laboratory of Biologically Active Substances, Department of Chemistry of Natural Compounds, University of Chemistry and Technology Prague, Prague, Czech Republic

**Keywords:** Hexahydrocannabinol, Cannabinoids, Pharmacokinetics, Psychophysical tests, Forensic toxicology, Mass spectrometry, Pharmacodynamics, Pharmacokinetics

## Abstract

The semi-synthetic cannabinoid hexahydrocannabinol (HHC) has become a highly discussed topic in forensic toxicology since 2022 due to its legal availability at this time and its psychoactive effects. This study aimed to investigate the pharmacokinetics, effects, and immunological detectability of HHC after oral (25 mg HHC fruit gum) and inhalative (three puffs from HHC vape) consumption with three participants per group. Serum (up to 48 h), urine (up to five days), and saliva (up to 48 h) samples were collected at different relevant time points and analyzed by HPLC-MS/MS for (9*R*)/(9*S*)-HHC, 11-hydroxy-HHC, and (9*R*)/(9*S*)-HHC carboxylic acid with a fully validated method. Additionally, immunological detectability was investigated with three different commercially available tests. To address the psychoactive effects, the subjective “high” feeling (scale 0–10) was monitored and different psychophysical tests (e.g. modified Romberg test, walk and turn) were conducted. Overall, the pharmacokinetics and effects of HHC were comparable to tetrahydrocannabinol (THC). However, the route of administration as well as inter-individual factors played a crucial role regarding maximum concentrations, pharmacokinetic profiles, and psychoactive effects.

## Introduction

The semi-synthetic cannabinoid hexahydrocannabinol (HHC) became popular as an alternative to delta-9-tetrahydrocannabinol (THC) in many countries since mid of 2022, as it has often not been covered by legal restrictions^1,2^. In the meantime, in most countries HHC is restricted by national laws^3–6^. However, HHC has been playing an increasing role in forensic toxicology laboratories for more than two years and the number of studies regarding HHC has been increasing distinctly since then^1,2,7^. In particular, various methods for the detection and quantification of HHC in human specimen have been published and the metabolism has been examined in more detail^7–10^. HHC and other semi-synthetic cannabinoids also gained importance in the author’s laboratory, particularly in cases involving driving under the influence and post-mortem investigations^7,11^. Consequently, this study focused on HHC to obtain more detailed data on its pharmacokinetics and psychoactive effects.

HHC exists in two enantiomeric forms, (-)- and (+)-HHC, whereby (+)-HHC does not occur naturally. (-)-HHC can occur as (9*R*) or (9*S*) diastereomer, whereby (9*R*)-HHC shows a significantly higher potency and efficacy than the (9*S*) diastereomer^12–15^. The metabolism of HHC appears to be similar to that of THC, resulting in the main metabolites 11-hydroxy-HHC (11-OH-HHC), 8-hydroxy-HHC (8-OH-HHC), and 11-nor-9-carboxy-HHC (HHC-COOH)^2,8,16^. Currently, research focuses particularly on the apparent stereoselectivity of the metabolism as well as on the identification of different main metabolites in blood and urine^8,17^.

In addition to HHC metabolism, further knowledge about its pharmacokinetics is important, especially in the context of forensic-toxicological investigations. New data from controlled consumption studies might improve the interpretation of HHC blood concentrations, e.g. regarding the classification of acute or subacute and occasionally or chronic consumption. A preliminary study from Di Trana et al. already highlighted concentrations in different human specimen, crucial pharmacokinetic parameters as well as the differences between both diastereomers after controlled inhalative consumption of HHC^17^.

Since HHC is a psychoactive component and the occurrence of HHC in samples from drivers under the influence (DUI) has been already reported in literature, a relevant impact on traffic safety can be assumed^7,18^. Therefore, the recognition of impaired drivers is of great importance. During traffic controls, mainly two approaches for identification of impaired drivers are conducted: psychophysical tests, which provide indications of failure symptoms, and immunological screening tests, which give a first indication of a potential drug consumption.

This preliminary study aimed at investigating general pharmacokinetic and psychophysical aspects of HHC as well as the detection of HHC consumption in context of traffic controls. For this explorative study a limited cohort of healthy volunteers was recruited and administered either three puffs from an HHC vape or a fruit gum containing 25 mg HHC. Main aim was to provide a general understanding of how HHC behaves in the body when consumed in a manner that reflects real-world usage. The focus laid on capturing concentration-time-curves for HHC and the metabolites 11-OH-HHC and HHC-COOH in different sample specimen after inhalative or oral consumption. Additionally, different psychophysical and immunological screening tests were evaluated for the detection of HHC consumption at various time points.

## Materials and methods

### Study participants

Three participants per study group were recruited. Inclusion criteria for study participation were a minimum age of 25 and a body mass index (BMI) between 18.5 and 29.9. Subjects were excluded from the study if residues from previous HHC consumptions could be detected in initial serum as well as urine samples. The participants did not report any chronic mental illnesses, diseases of the heart, lungs, gastrointestinal tract, liver or kidneys as well as any acute diseases. Further exclusion criteria for female participants were pregnancy (checked using a commercially available pregnancy test immediately before administration of HHC) and breastfeeding. All participants confirmed that they met the respective criteria. Additional information about the participants are listed in Table 1.

**Table 1:** Basic information about study participants.

Group	Abbreviation	Gender	Age, years	Weight, kg	Height, cm	Experience with HHC	Experience with THC
Oral	O1	Female	26	88	174	Once	Rarely
	O2	Male	30	71	182	No	Once
	O3	Female	29	58	159	No	No
Inhalative	I1	Female	29	80	172	Rarely	Regularly
	I2	Male	25	90	195	Rarely	Regularly
	I3	Male	27	69	184	No	Once

### Ethics

The study was approved by the ethics committee of the medical faculty of the University of Leipzig (370/23-ek). It was performed in accordance with the Declaration of Helsinki and all relevant guidelines/regulations. Informed consent for the conduction of the study and publication of the results was obtained from all participants.

### Study design

The study was non-blinded and not randomized. In the oral consumption group, one HHC fruit gum was consumed. In the inhalative consumption group, three puffs were taken from an HHC vape. The dosages were chosen based on usual recommendations for inexperienced consumers according to literature data from other studies^8,17^ as well as customer reports from internet forums, since only limited information and sources for HHC dosage were available. They are classified in the low range to prioritize safety and minimize adverse effects while ensuring measurable concentrations and psychoactive effects. Serum, urine, and saliva were collected on different time points (see Supplemental Table S1). The serum samples on the first day were taken using a peripheral intravenous catheter. A regular blood sampling procedure was conducted after 24 h and 48 h. Different immunological screening tests for serum, urine, and saliva have been performed. The subjective “high” feeling of the participants was queried on different time points using a unitless scale of 0 to 10 (0 = no “high” feeling; 10 = maximum “high” feeling). In addition, standardized psychophysical tests were carried out at several time points.

### HHC products

Both HHC products were from Cannastra (Příbram, Czech Republic) and bought in a local shop (Leipzig, Germany) for hemp products. Prior to the study, both products were analyzed for their HHC concentration applying the HPLC-MS/MS method described below. For this purpose, they were dissolved, diluted with acetonitrile (ACN), and then processed according to the sample preparation protocol described below. The distillate in the vape consisted of nearly 100% HHC, corresponding to 1 mg HHC per 1 mg distillate. One fruit gum contained an absolute dose of 25 mg HHC. All fruit gums used in the study were from the same batch. (9*R*)-HHC and (9*S*)-HHC were present in both products with amounts of 78 and 22%, respectively. No residual THC and other popular cannabinoids were detected.

### Biological materials

Authentic blank serum (S-Monovette^®^ 9 mL serum-gel, Sarstedt, Nümbrecht, Germany), urine, and saliva for method development and validation were obtained from healthy volunteers. Specimen of serum, urine, and saliva were collected from the study subjects following the scheme in Supplemental Table S1.

### Reagents and chemicals

LC-MS grade ethyl acetate, n-hexane, water, ACN, and methanol (MeOH) were obtained from VWR International (Darmstadt, Germany). Acetic acid was from Carl Roth (Karlsruhe, Germany). Solutions of THC-D3, 11-OH-THC-D3, and THC-COOH-D3 with concentrations of 100 µg/mL in MeOH as well as ß-glucuronidase from *Patella vulgata* (aqueous solution, ≥ 85,000 units/mL) were purchased from Sigma-Aldrich (Steinheim, Germany). Solutions of (9*R*)-HHC, (9*S*)-HHC, and (9*R*)-HHC-D9 in ACN (1 mg/mL) as well as crystalline (9*R*)-HHC-COOH, (9*S*)-HHC-COOH, (9*R*)-11-OH-HHC, and (9*S*)-11-OH-HHC were from Cayman Chemical (Biomol, Hamburg, Germany). Stock solutions of the crystalline analytes (1 mg/mL) as well as different dilutions for further use were prepared in ACN.

### Calibrators and quality control (QC) samples

Matrix-matched calibration samples were prepared by spiking blank pooled specimen (serum, urine or saliva) of five volunteers. Concentrations of the calibration levels are shown in Supplemental Table S2. Quality control (QC) samples for all analytes in two different concentrations (low, high) were prepared by spiking blank pooled specimen (see Supplemental Table S3 and S4).

### HPLC-MS/MS analysis

#### Sample preparation

For sample preparation, 200 µL specimen (serum, urine, saliva), 10 µL internal standard mixture (final concentrations: 10 ng/mL (9*R*)-HHC-D9, THC-D3 and 11-OH-THC-D3, 50 ng/mL THC-COOH-D3), 100 µL acetic acid (1.5% in water), and 5 µL of ß-glucuronidase were incubated for 2 h on a shaker at 37 °C. Afterwards, the mixture was extracted with 1000 µL hexane/ethyl acetate (80/20, v/v) for 5 min on a shaker. After 5 min centrifugation (13000 rpm) the supernatant was evaporated in a glass vial under nitrogen. The residue was resuspended in 25 µL ACN and 25 µL water.

#### Instrumentation and measurement method

The analysis was utilized on an Agilent 1290 Infinity II LC system (Agilent Technologies, Waldbronn, Germany) coupled with an ABSciex QTrap 5500 mass spectrometer, controlled by Analyst^®^ 1.7.1 software (ABSciex, Darmstadt, Germany). Chromatographic separation was conducted using a Gemini^®^ 3 μm NX-C18 110 Å 150 × 3 mm analytical column (Phenomenex, Aschaffenburg, Germany). Eluent A consisted of 1.5 mL 85% formic acid in 1000 mL water, while eluent B contained 1.5 mL 85% formic acid in 1000 mL ACN. The gradient profile was as follows: 0–1 min at 50% B, 1–16 min ramping to 100% B, 16–17.5 min holding at 100% B, 17.5–19 min holding at 2% B, 19–20 min holding at 50% B, with a total runtime of 20 min. The flow rate was maintained at 0.4 mL/min. Column oven temperature was set at 30 °C. The injection volume was 5 µL.

Ionization of analytes was achieved using positive-mode electrospray ionization with nitrogen as curtain gas at 35 psi and an ionspray voltage of 4500 V. The temperature of the ion source was maintained at 600 °C. Ion source gases 1 and 2 were set at pressures of 50 psi and 70 psi, respectively. The parameters for the scheduled multiple reaction monitoring (sMRM) method are detailed in Supplemental Table S5. These included an entrance potential of 10 V for all analytes. The MRM detection window was 50 s with a target scan time of 0.5 s per sMRM experiment.

#### Method validation

The method was developed for the targeted analysis of various cannabinoids and their metabolites (see Supplemental Table S5). It was validated for the quantitative analysis of (9*R*)-/(9*S*)-HHC, 11-OH-HHC, and (9*R*)-/(9*S*)-HHC-COOH in serum based on the guideline of the German Society of Toxicological and Forensic Chemistry (GTFCh). For serum, limits of detection (LOD), lower limits of quantification (LLOQ), accuracy, intra-day precision, inter-day precision, processed sample stability, freeze-thaw-stability, storage stability, matrix effects and recovery were validated. For urine and saliva specimens, matrix matched calibrations were set up and LOD, LLOQ, accuracy, intra-day precision, and inter-day precision were determined as part of a short cross-validation.

The peak area ratios from analytes to internal standards were plotted against the concentrations to obtain calibration curves. (9*R*)-HHC-D9 was used as internal standard for (9*R*)-/(9*S*)-HHC, 11-OH-THC-D3 for 11-OH-HHC, and THC-COOH-D3 for (9*R*)-/(9*S*)-HHC-COOH. LODs (S/*N* = 3) were calculated based on six spiked samples at the lowest calibration point for each analyte. LLOQs were determined according to the guideline of the GTFCh (alternative method 2) using bias and precision data (required to be ≤ 20% each)^19^.

The determination of intra-day and inter-day precision was performed by the analysis of samples of each QC concentration ten times as well as on ten consecutive days, respectively. Accuracy was calculated based on ten inter-day samples. Processed sample stability at room temperature over three days, freeze-thaw-stability in serum over three cycles and storage stability in serum at −20 °C over six weeks were determined for both QC concentrations. Analytes were considered as stable if concentration change in comparison to reference samples was ≤ 20%. Matrix effects and recovery for serum were calculated based on five different samples spiked pre-extraction, five samples spiked post-extraction, and five neat standard solutions for both QC concentrations using Valistat 2.00.1 (Arvecon, Walldorf, Germany).

### Pharmacokinetic investigations

One serum, urine, and saliva sample from each sampling time point and each participant was processed and analyzed as described above. Maximum concentration (c_max_) and time to reach maximum concentration (t_max_) in serum were determined for each participant. Elimination constant (k_e_) and elimination half-life (t_1/2_) of the analytes in serum were calculated using a non-compartmental analysis in the PKanalix 2024 software (Lixoft, Antony, France) applying Eq. (1).

1 $${t_{\frac{1}{2}}}=~\frac{{{\text{ln}}\left( 2 \right)}}{{ - ~slope}}$$

For compartmental analysis the measured data were fitted in Wolfram Mathematica 12 (Wolfram Research, Champaign, USA) using the two-compartment model with first-order absorption.

Creatinine concentrations used to normalize urine concentrations of the analytes were determined in an external, accredited laboratory. Serum concentrations as well as urine concentrations normalized for creatinine were plotted against time using IBM SPSS Statistics 29.0 (IBM, Armonk, New York, USA).

### Psychophysical investigations

Different tests for the investigation of psychophysical effects and the prediction of potential impairments in road traffic were conducted according to commonly used tests of the Saxon police. These included firstly objective tests such as the modified Romberg test, the post-rotational nystagmus test (PRN), and the assessment of the pupil size, which were conducted on multiple time points (around 30 min, 1 h, 2 h, 4 h, 6 h, and 8 h after consumption). Additionally, non-objective/learnable tests such as walk and turn (WAT), one leg stand (OLS), finger-to-finger (FTF) test, and finger-to-nose (FTN) test were conducted only once, if possible in the acute phase, when the participants reported the most intensive subjective “high” feeling. The single tests are briefly described in the following.

#### Modified romberg test

The subjects were instructed to stand with their feet together and arms at their sides. They should maintain this position while the instructions were given. After the start signal, the participants should tilt their head back and close their eyes. This position was demonstrated by the instructor, but without closing the eyes. When the subjects estimated that 30 s have passed, they should return their head to a normal position, open their eyes, and say “stop”. The recommendation to count to 30 was not provided, but it was also not prohibited. The participants were asked how long they maintained that position.

Potential irregularities in this test were: deviations from estimated to the actual time span of more than ± 5 s, tremor of the whole body or eyelids, slack posture (low muscle tone), swaying of the head or the whole body, leaving the starting position^20^.

#### Post-rotational nystagmus test (PRN)

The subjects rotated around their own body axis 5 times within 10 s, then fixed their gaze on the examiner’s index finger, which was about 25 cm away. The time until the nystagmus of the eye stopped was noted. Values above 10 s were considered as abnormal^21^.

#### Pupil size

The pupil size was assessed with a forensic ruler under consistent room light conditions. Diameters between 3 and 9 mm were assessed to be normal.

#### Walk and turn test (WAT)

WAT test was conducted according to the standardized field sobriety testing procedures^22^. Failure of the test was noted if the participant made two or more of the following errors: no balance during instructions, started walking before instructions were completed, stopped walking, missed heel-to-toe, left the straight line, used arms for balance (more than 15 cm away from body), turned improperly, wrong number of steps^22^.

#### One leg stand (OLS)

OLS test was conducted according to the standardized field sobriety testing procedures^22^. It was considered abnormal if two or more of the following signs were observed: swaying while balancing, using arms for balance, hopping, putting the raised foot down, difficulties while counting^22^.

#### Finger-to-finger test (FTF)

In FTF test, with closed eyes, the arms had to be stretched out horizontally to the sides and the index fingertips had to be slowly brought together in front of the nose with the arms stretched out. Severe deviation of the fingertips or trembling of the fingers were considered as failure.

#### Finger-to-nose test (FTN)

The starting position involved standing with feet together, arms resting by the sides, head tilted backward, and eyes shut. Then the subjects had to point the tip of their index finger to the tip of their nose following the instructions. The order was precisely specified: left – right – left – right – right – left^23^. Failures included missing the tip of the nose, using the wrong arm or the wrong finger. Attention was also paid to noticeable body tremors or impaired fine motor skills.

### Evaluation of immunological screening tests

For detection in saliva, DrugWipe^®^ 5 S tests (Securetec, Munich, Germany) were used. SureStep™ Multi-Drug Urine tests (Innovacon Inc., San Diego, USA) were applied for the evaluation of the detection in urine samples. The THC Direct ELISA Kit (Abbott Immunalysis, Pormona, USA) combined with a Tecan hydro flex washer, a Tecan sunrise plate reader, and the software Magellan™ (Tecan Group, Männedorf, Switzerland) were used for serum. All tests were performed in accordance with the manufacturer’s instructions. Immunological tests for urine and serum were conducted on each sampling time point. Saliva tests were performed at all time points as presented in Supplemental Table S1 (marked with an asterisk).

## Results and discussion

### Method validation

Chromatographic separation could be achieved for all analytes and diastereomers except for (9*R*)- and (9*S*)-11-OH-HHC as shown in Supplemental Fig. S1. The essential validation data for serum are detailed in Supplemental Table S3. LOD, LLOQ, accuracy as well as intra- and inter-day precision in serum are comparable or even better than literature data^18,24^. Recovery and matrix effects met the criteria in the Valistat software according to the GTFCh guideline^19^. Stability experiments were conducted solely for serum. All analytes except 11-OH-HHC were stable in processed-samples over three days indicated by concentration changes between − 5.3% and + 3.3% after three days compared to freshly prepared samples. 11-OH-HHC showed a decrease of concentrations of about 20% after two days, therefore immediate measurements after sample processing and short batches were ensured. All analytes were stable over three freeze-thaw cycles, indicated by concentration changes between −7.0 and +2.9%. Stability in serum samples during storage at −20 °C over at least six weeks could be confirmed for all analytes (concentration changes: 3.9–12.3%). Due to the successful extensive validation for serum and the appropriate LOD, LLOQ, precision, and accuracies (see Supplemental Table S4) for urine and saliva matrix, the method was considered appropriate for the study design.

### Pharmacokinetic investigations

#### Serum

Essential pharmacokinetic data for all participants and analytes in serum are summarized in Table 2.

**Table 2:** Pharmacokinetic data in serum for all analytes and individuals.

		(9R)–HHC	(9S)–HHC	11–OH–HHC	(9R)–HHC–COOH
O1	c_max_, ng/mL	12.0	4.39	9.49	37.1
	t_max_, h	2	2	2.5	2.5
	k_e_	0.41	0.49	0.35	0.0072
	t_1/2,_ h	1.70	1.42	1.97	96.1
O2	c_max_, ng/mL	7.07	2.47	5.84	10.7
	t_max_, h	1.25	1.25	2.25	8
	k_e_	–	–	–	–
	t_1/2,_ h	–	–	–	–
O3	c_max_, ng/mL	2.75	1.07	3.66	13.4
	t_max_, h	1.75	1.75	3	2.75
	k_e_	0.25	0.36	0.24	0.011
	t_1/2,_ h	2.78	1.94	2.87	61.8
I1	c_max_, ng/mL	15.8	5.93	0.93	1.66
	t_max_, h	0.05	0.05	0.58	0.25
	k_e_	0.46	0.59	0.27	0.010
	t_1/2,_ h	1.52	1.18	2.58	66.2
I2	c_max_, ng/mL	179	61.3	3.88	13.6
	t_max_, h	0.10	0.05	1	0.45
	k_e_	0.35	0.36	0.27	0.0086
	t_1/2,_ h	1.97	1.95	2.56	81.0
I3	c_max_, ng/mL	142	48.2	4.76	10.5
	t_max_, h	0.033	0.033	0.10	0.15
	k_e_	0.33	0.37	0.26	0.020
	t_1/2,_ h	2.11	1.87	2.64	35.4

**Fig. 1:**
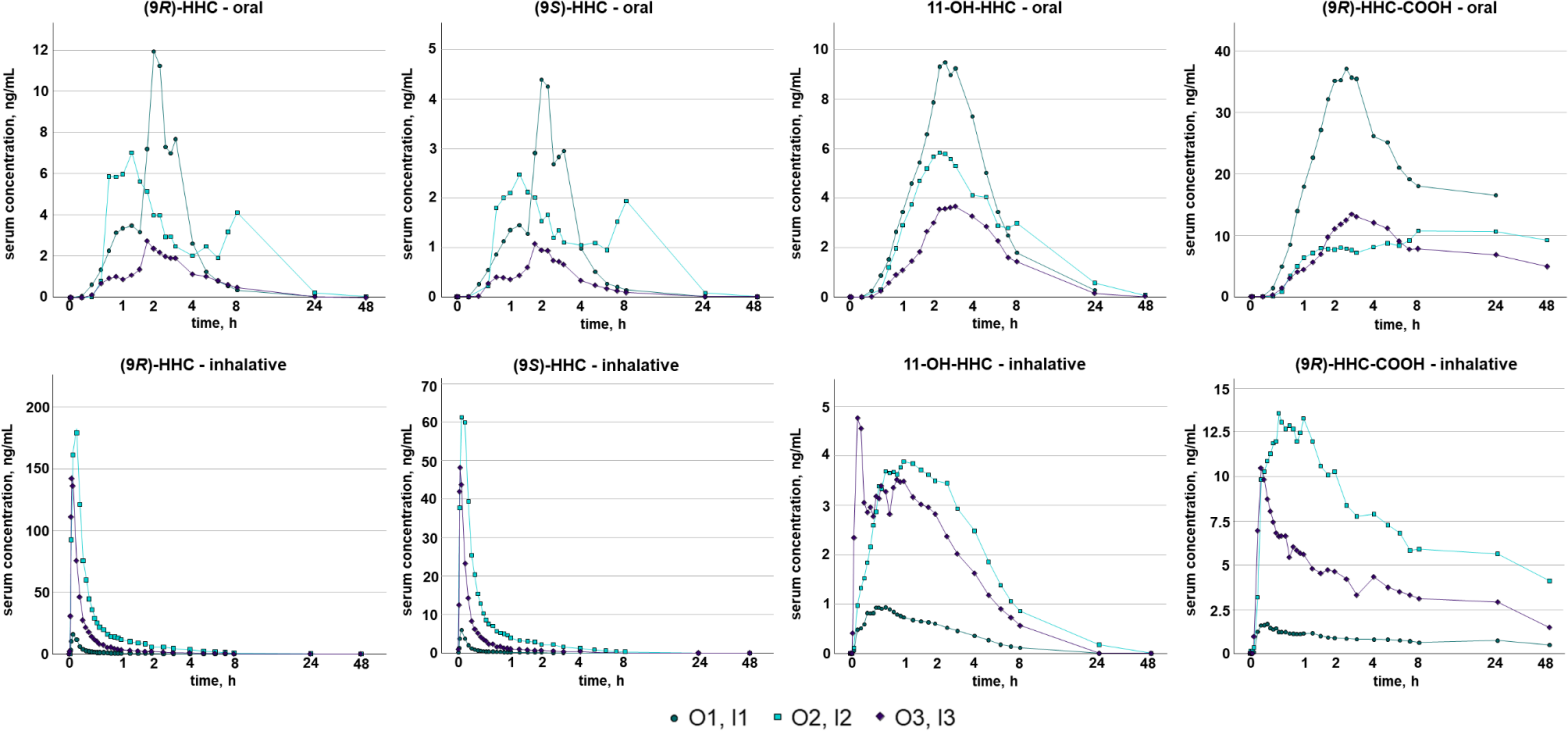
(9*R*)–HHC, (9*S*)-HHC, 11–OH–HHC, and (9*R*)–HHC–COOH concentrations in serum in relation to time after oral (top) and inhalative (bottom) consumption.

Both HHC diastereomers were detectable in serum of all participants. The corresponding concentration-time-curves are shown in Fig. 1. The maximum concentrations after oral consumption of 25 mg HHC were reached after 1.25–2 h and ranged from 2.75 to 12.0 ng/mL for (9*R*)-HHC as well as from 1.07 to 4.39 ng/mL for (9*S*)-HHC. These concentrations were comparable to concentrations after inhalative consumption of the same dose in the study of Di Trana et al. (mean ± SD, (9*R*)-HHC: 7.9 ± 7.3 ng/mL, (9*S*)-HHC: 2.3 ± 1.3 ng/mL).^17^ The maximum HHC concentrations after inhalative consumption were reached within 2–6 min with values from 15.8 to 179 ng/mL for (9*R*)-HHC and 5.93–61.3 ng/mL for (9*S*)-HHC. Due to inter-individual differences in inhalation technique, no precise statement about the absolute dose in the inhalation group can be made, so a direct comparison of the maximum concentrations with literature data is not feasible.

In all samples, the (9*R*)-HHC concentration was higher than the (9*S*)-HHC concentration, with a median ratio of 2.62 (range: 1.97–5.70) after oral and 3.02 (range: 2.37–3.67) after inhalative consumption. Overall, the ratios corresponded to literature data from driving under the influence of HHC cases (range: 1.62–2.80)^7^ as well as to another pharmacokinetic study from Di Trana et al. (average (9*R*)-HHC concentration 3-fold higher compared to (9*S*)-HHC)^17^. The (9*R*)-/(9*S*)-HHC ratio in serum does not seem to be related to the ratio of the product consumed, since ratios from the presented study are comparable to the study of Di Trana et al., even though they used a product with a ratio of 50/50 compared to 78/22 in this study^17^. Additionally, significant differences of medians and distributions (Median test and Mann-Whitney-U-test, both *p* < 0.001) between oral and inhalative consumption were pronounced, despite both HHC products containing the same R/S ratio. Within the oral consumption group, no significant (median test; *p* ≥ 0.05) differences between the participants were observed. However, in the inhalative group, significant differences between participants I2 and I1 (median test; *p* = 0.002) as well as participants I2 and I3 (median test; *p* < 0.001) were pronounced. A potential reason for the differences in the ratios might be different metabolism patterns, depending on the way of consumption (oral or inhalative). Additionally, inter-individual differences in metabolism resulting from cytochrome P450 variations and other individual factors might play a crucial role.

The t_max_ values of HHC corresponded to literature data from THC/marihuana consumption for the respective way of consumption^25,26^. The median t_1/2_ (*n* = 5) for (9*R*)-HHC and (9*S*)-HHC were 1.97 h (range: 1.52–2.78 h) and 1.87 h (range: 1.18–1.95 h), respectively. No relevant difference between oral and inhalative consumption regarding the half-life was observed. Compartmental analysis resulted in the best fit using the two-compartment model with first-order absorption (see Supplemental Fig. S2 and S3). Interestingly, in participant O2, a second absorption phase was visible, resulting in an increase of the HHC concentrations after 6 h. Since no restrictions regarding food intake during the study were made, an influence on the absorption of HHC could not be excluded. Due to this second absorption no pharmacokinetic fit and therefore no calculation of k_e_ and t_1/2_ was feasible for O2.

The time to reach the maximum 11-OH-HHC concentrations in serum appeared to be longer compared to the time when the maximum HHC levels were determined. The maximum concentrations of 11-OH-HHC after oral and inhalative consumption ranged between 3.66 and 9.49 ng/mL and 0.93–4.76 ng/mL, respectively. After oral as well as inhalative consumption, a distinct plateau of the 11-OH-HHC concentrations was formed, resulting in a median t_1/2_ of 2.58 h (range: 1.97–2.87 h), which is distinctly longer compared to the HHC diastereomers. The metabolite (9*R*)-HHC-COOH also appeared to form a very long state of equilibrium, similar to what is known from THC-COOH^27^, resulting in a median t_1/2_ of 66.2 h (range: 35.4–96.1 h). The maximum concentrations varied between individuals (oral: 10.7–37.1 ng/mL; inhalative: 1.66–13.6 ng/mL). Only very small amounts of (9*S*)-HHC-COOH were detectable in serum samples (LOD: 0.08 ng/mL) from both groups, which corresponds to results by Kronstrand et al., who also found only the (9*R*)-HHC-COOH diastereomer in blood samples^18^. Interestingly, the maximum concentrations of the metabolites were distinctly higher after oral consumption, even if the maximum concentration of the HHC diastereomers were lower compared to inhalative consumption. Potential reasons for this may be the different absorption mechanism, absolute dose, first-pass effect, and individual metabolic factors. Additionally, inter-individual differences in the concentrations seems to be much more pronounced after inhalative consumption, certainly mainly due to variation in inhalation techniques resulting in different absolute doses of HHC.

#### Urine

**Fig. 2:**
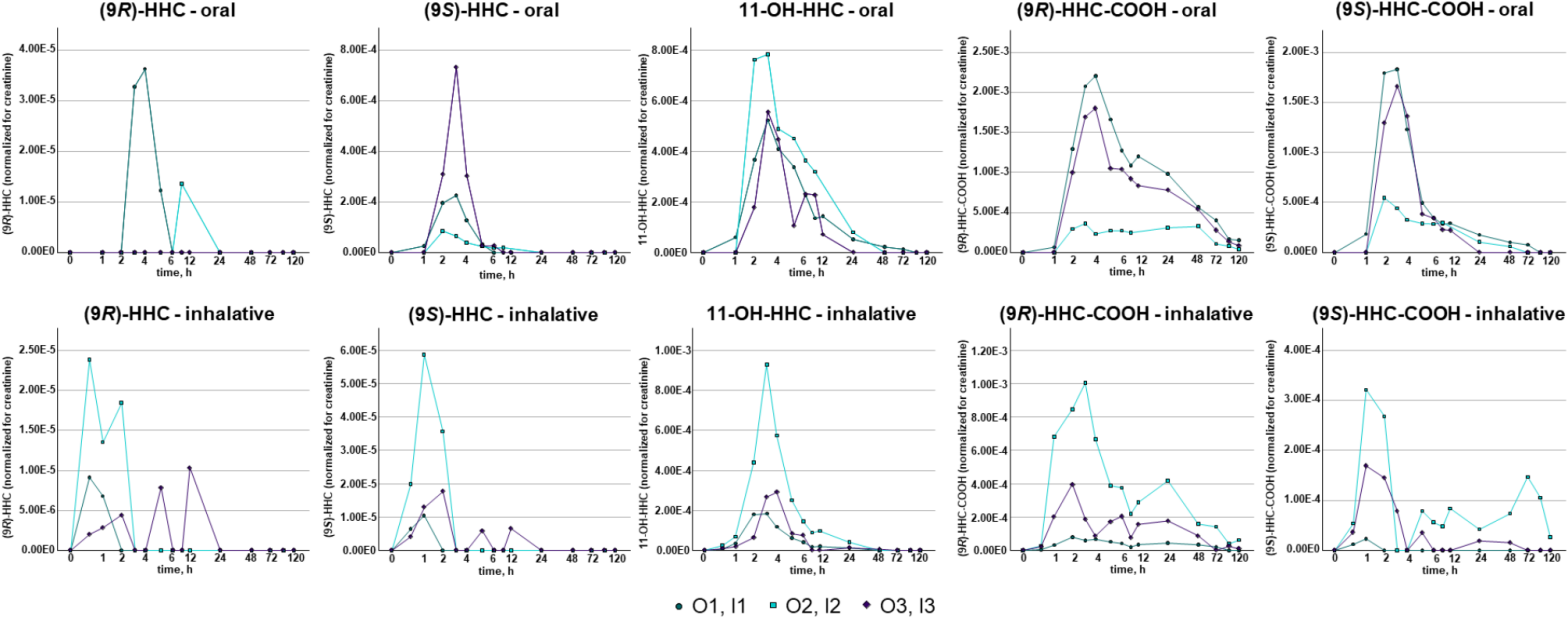
(9*R*)–HHC, (9*S*)–HHC, 11–OH–HHC, (9*R*)–HHC–COOH, and (9*S*)–HHC–COOH concentrations in urine normalized for creatinine in relation to time after oral (top) and inhalative (bottom) consumption.

Figure 2 shows the urine concentrations of the analytes normalized for creatinine in relation to time. In urine, both HHC diastereomers were detectable in each individual at certain time points, albeit in very low amounts. This slightly contrasts to findings of Schirmer et al., who detected (9*R*)-HHC only after inhalative consumption in urine^8^. However, as previously mentioned, (9*R*)-HHC was only detectable in very small amounts in this study. In addition, the oral dose used here was a little higher compared to Schirmer et al. (25 mg vs. 20 mg^8^), which may lead to concentrations slightly above the LOD. Unfortunately, the study of Schirmer et al. contains no statements about detection limits and concentrations of the HHC diastereomers and included only two participants, which makes it difficult to compare the data.

11-OH-HHC was also detectable in the urine of all participants with the highest concentrations around 2–4 h after consumption. In contrast to serum, (9*S*)-HHC-COOH was detectable in urine samples from all participants at concentrations between < LLOQ and 8.92 ng/mL compared to concentrations between < LLOQ and 20.2 ng/mL for (9*R*)-HHC-COOH, indicating considerable metabolic differences between the two diastereomers. This differs to literature data from Di Trana et al., who found (9*S*)-HHC-COOH only in blood, but not in urine^17^. A possible reason might be a difference between serum and whole blood. Additionally, the slightly lower LOD for (9*S*)-HHC-COOH in urine in this presented study (0.25 ng/mL) compared to the method used by Di Trana et al. (0.50 ng/mL) might be an explanation, since the measured concentrations were predominantly within this particular concentration range. The ratios of the metabolites in urine differed distinctly between the participants, both in oral as well as inhalative group, which may indicate inter-individual differences in metabolism. Interestingly, in contrast to serum, no differences in t_max_ between the consumption groups were observed in urine.

#### Saliva

In saliva, only (9*R*)-HHC and (9*S*)-HHC but no metabolites were detectable, which corresponds to literature data^17,28^. For all participants, maximum concentrations of the HHC diastereomers were measured directly after consumption, presumably caused by residues in the mouth. Concentrations decreased rapidly in all subjects, with maximum detection times via LC-MS/MS in saliva of 8 h after oral consumption and 5 h after inhalative consumption. However, it must be noted that matrix effects were very high and also fluctuating between the individual saliva samples. Therefore, the LODs for saliva are significantly higher than for serum and urine. Methods including more sensitive LC-MS/MS devices or more complex purification steps in sample preparation, such as solid-phase-extraction, could lead to significantly longer detection times.

### Psychophysical investigations

**Fig. 3:**
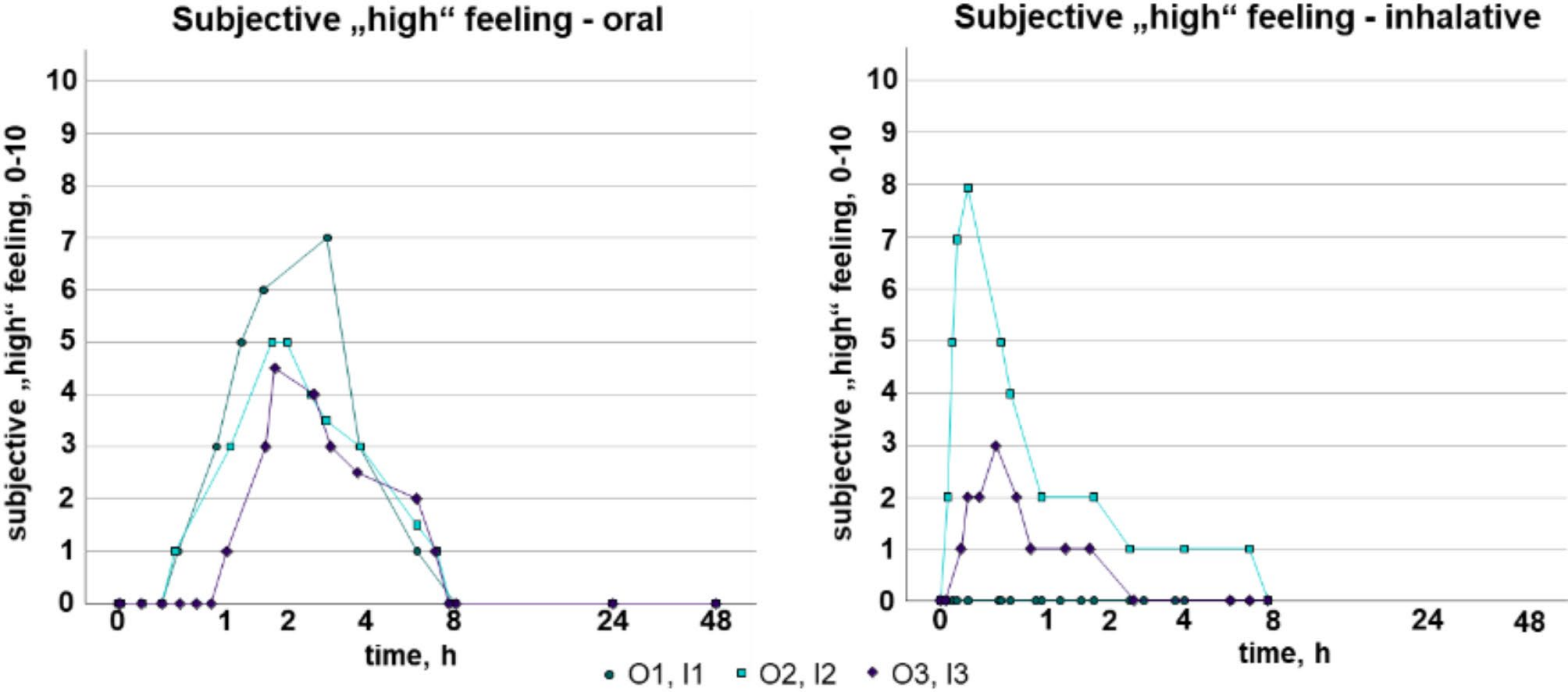
Time course of the subjective “high” feeling in the oral (left) and inhalative (right) consumption group.

With the exception of subject I1, all participants reported a psychoactive effect. Additionally, all six participants communicated a dry mouth. The maximum subjective “high” feeling ranged between 4.5 and 7 in the oral as well as between 0 and 8 in the inhalative consumption group (see Fig. 3) on the unitless scale from 0 to 10. As previously reported for the HHC concentrations, also the subjective “high” feeling showed greater inter-individual differences after inhalative compared to oral consumption, presumably due to the different inhalation techniques.

**Fig. 4:**
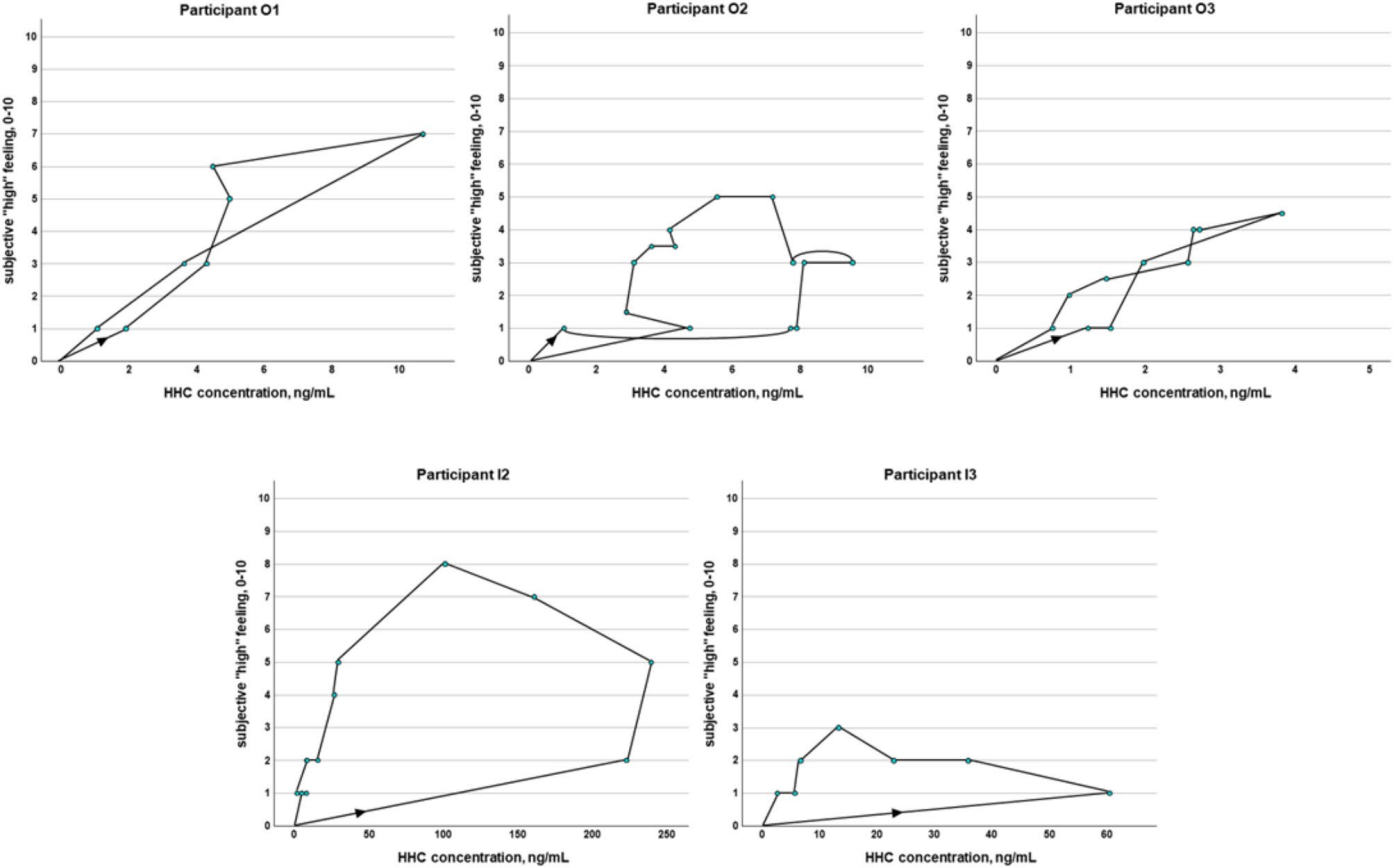
Correlation between subjective “high” feeling (y-axis), HHC concentrations (x-axis), and time course of the single measurements (marked by black line and arrow) in the oral consumption group (top) and in the inhalative consumption group (bottom): maximum “high” feeling simultaneously with maximum concentration in participants O1 and O3; maximum “high” feeling only in elimination phase in participants O2, I2, and I3.

Overall, the courses of the feeling-time-curves (see Fig. 3) were similar to the concentration-time-curves for HHC (see Fig. 1). However, only in participants O1 and O3, the maximum subjective “high” feeling was reached together with the highest concentration (see Fig. 4). In participants O2, I2, and I3, the maximal effect was reached delayed compared to the maximum concentrations (see Fig. 4), which corresponds to literature data for THC^29,30^. The more heterogeneous plots of the feeling-concentration curves after oral consumption compared to inhalation consumption might be explained by the delayed and food-dependent absorption and thus effect. However, it should be noted, that not for every serum sampling point a corresponding subjective “high” feeling was monitored, which might have led to different curves. However, the subjects were able to report at any time if a reportable change in the “high” feeling occurred.

Results from the psychophysical tests are summarized in Table 3. All subjects showed slight abnormalities in the tests even prior to consumption (O1: PRN of 12 s; other participants: more than 5 s deviation in modified Romberg test). No further abnormalities after consumption were observed in any of the subjects regarding pupil size and PRN. Participant O1 showed slight uncertainties in WAT, OLS, and FTN, conducted around 35 min after consumption. Subject I1 only missed by 3 cm in FTF, but showed no further abnormalities. The irregularities in participants O1 and I1 would presumably not have been considered as a failure of the tests resulting in an immunological drug test in a traffic control. However, especially in participant O1, the non-subjective tests were unfortunately carried out too early and not in the phase of the maximum “high”-feeling, since the participant felt impaired and it was not foreseeable how much that feeling would increase. Therefore, it can be assumed that the abnormalities in the tests would have been more significant at a later time point.

**Table 3:** Results of psychophysical tests and subjective feelings of the participants; *B.c.* before consumption, *A.c.* after consumption.

	Modified romberg test	Post-rotational nystagmus test	Pupil size	Walk and turn	One leg stand test	Finger-to-finger test	Finger-to-nose test	Maximum subjective “high” feeling	Other sensations
O1	b.c.: 30 s a.c.: 28–36 s	b.c.: 12 s a.c.: 8–12 s	b.c.: 4 mm a.c.: 4 mm	Swayed once	Subtle, whole-body tremors; used arms for balance	No abnormalities	Missed once by 2 cm	7	Dry mouth; first relaxed, then panicked
O2	b.c.: 40 s a.c.:23–32 s	b.c.: 6 s a.c.: 4–6 s	b.c.: right 4 mm, left 3 mm a.c.: right 4–5 mm, left 3–4 mm	Swayed twice; stepped to the side; used arms for balance	No abnormalities	Missed by 1 cm	Rejected due to peripheral intravenous catheter	5	Dry mouth; similar to mild drunkenness
O3	b.c.: 17 s a.c.: 24–26 s	b.c.: 6 s a.c.: 4–6 s	b.c.: 5 mm a.c.: 4–5 mm	Swayed once	Swayed; put the raised foot down; difficulties while counting	No abnormalities	No abnormalities	4.5	Dry mouth; similar to mild drunkenness
I1	b.c.: 39 s a.c.: 25–35 s	b.c.: 6 s a.c.: 6–7 s	b.c.: 3 mm a.c.: 3 mm	No abnormalities	No abnormalities	Missed by 3 cm	No abnormalities	0	Dry mouth
I2	b.c.: 36 s a.c.: 26–28 s	b.c.: 7 s a.c.: 6–8 s	b.c.: 3 mm a.c.: 3 mm	Had to leave starting position; moderate trembling of legs; used arms for balance	Swayed; used arms for balance	No abnormalities	Slight swaying in all directions	8	Dry mouth; pressure on forehead and ears
I3	b.c.: 40 s a.c.: 22–31 s	b.c.: 7 s a.c.: 6–7 s	b.c.: 4 mm a.c.: 4 mm	No abnormalities	Swayed; difficulties while counting	No abnormalities	No abnormalities	3	Dry mouth; similar to mild drunkenness

In contrast, participants O2 and O3 showed striking abnormalities in the tests conducted around 2 h post-consumption, indicating an impairment, especially in WAT and OLS, respectively. Also participants I2 and I3 showed severe impairments in those tests, conducted around 40 min after inhalation. The first attempt to carry out the tests even had to be aborted in both cases due to severe dizziness (10 min after consumption).

No or only slight impairments were observed in FTN and FTF for all participants, indicating a reduced suitability of these tests for the detection of HHC consumption.

Overall, the psychoactive sensations as well as impairments are comparable to known data after cannabis consumption^31^. This corresponds to literature data which indicated the similarity of the effects between HHC and THC^2^. However, as well as the concentrations, also the effects, especially in the inhalative consumption group, differed greatly, although participants consumed the same subjective dose of three puffs. This once again shows that consumption statements do not have significance, since inhalation, absorption as well as effects have wide inter-individual fluctuations. Additionally, it can be assumed, that as previously described for THC, no direct correlation between HHC concentration and psychomotor skills exists^31^.

### Evaluation of immunological screening tests

**Fig. 5:**
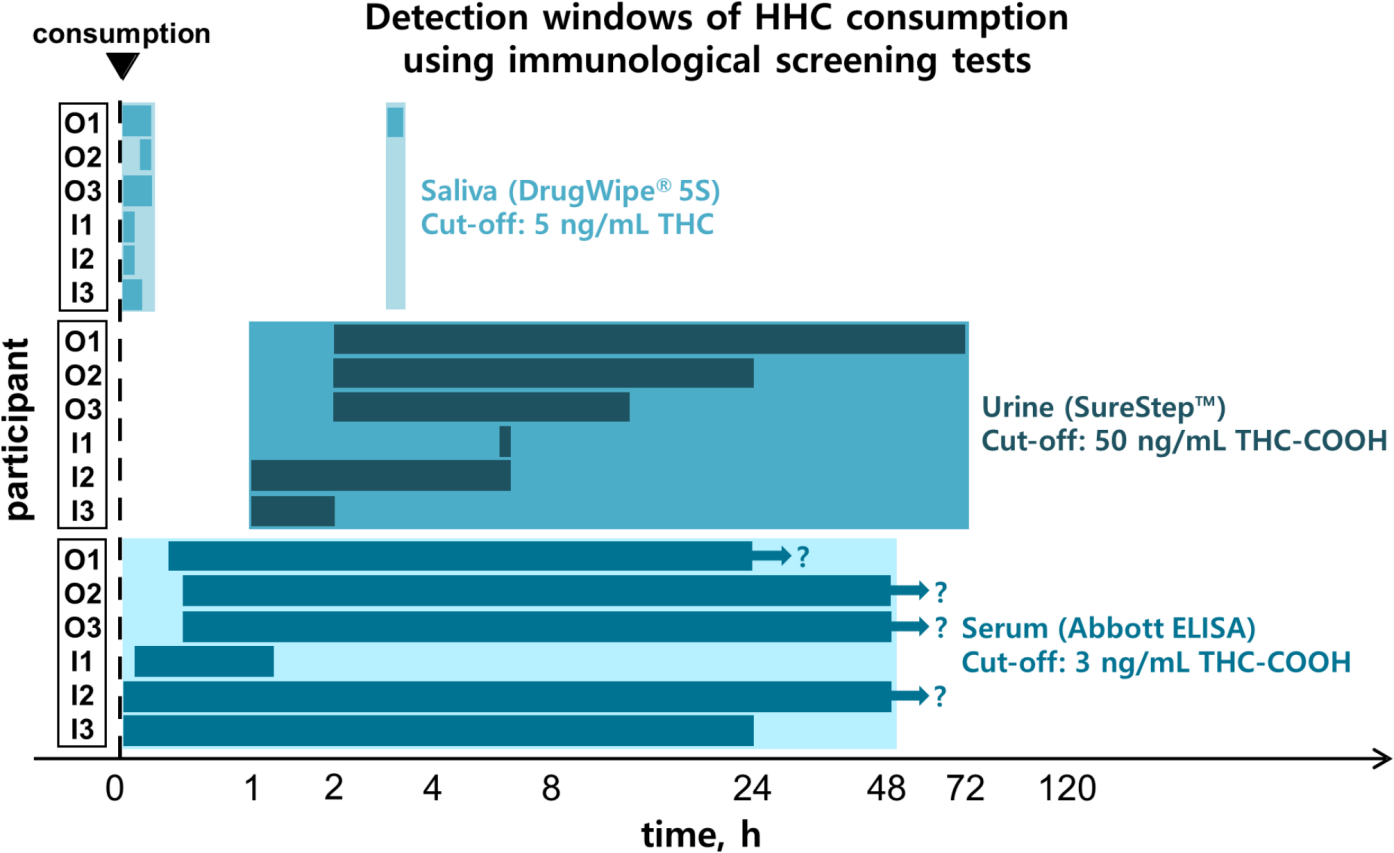
Detection windows of HHC consumption using immunological screening tests for saliva (DrugWipe^®^ 5 S), urine (SureStep™), and serum (Abbott ELISA).

Immunological screening tests are crucial tools to detect drug consumption in traffic besides psychophysical tests. Despite their initial goal to detect classic drugs such as THC, amphetamines, cocaine, or opiates, they can have cross-reactivities with other drugs due to structural similarities. Previous preliminary studies have shown that HHC, 11-OH-HHC, and HHC-COOH may also exhibit some cross-reactivity with immunological tests^7,18,32,33^. However, so far only spike experiments have been conducted with the following disadvantages: no investigation of combined cross-reactivity of different metabolites, no depiction of authentic concentrations as well as potential influence of the organic solvent in the spiked sample on the immunological tests. This study therefore aimed to determine cross-reactivities after controlled HHC consumption. All tests were negative prior to consumption for all participants. The results are summarized in Fig. 5.

The DrugWipe^®^ 5 S tests for saliva were positive in all cases in a time interval of up to 10 min after consumption. Initially, these tests were developed for the detection of THC with a cut-off of 5 ng/ml in saliva. The cross-reactivities with HHC were already shown with spike experiments in literature^7,32^. Positivity thresholds of 250 ng/mL for (9*R*)-HHC^7,32^ and 500 ng/mL for (9*S*)-HHC^32^ were reported, which is consistent with the positive results immediately after consumption and the associated measured concentrations in this study. However, the initial positive results are presumably only due to residues of HHC in the mouth. Only in case O1, the saliva test turned positive again 3 h post-consumption. At this time point, the HHC concentrations measured in saliva via HPLC-MS/MS were already below the threshold determined in the spike experiments. A reason for the positive result might be a combined cross-reactivity of different metabolites that were not investigated in our study. However, also a false positive result due to interactions with food or drink residues might be possible. As a limitation it has to be mentioned, that due to limited availability of the tests not on all saliva sampling points an immunological test was conducted. Additionally, no specifications for drinking and eating during the study were made, so potential dilution effects cannot be excluded with certainty.

The SureStep™ Multi-Drug Urine test was initially designed for the detection of THC-COOH in urine with a cut-off of 50 ng/mL. An expected cross-reactivity with HHC-COOH was already proven in literature^7^. The test was positive for all participants. However, for participant I1 it was only positive in one sampling point after 6 h. For participant I2, it was positive from 1 h to 6 h, but had a negative result in the sample collected after 3 h. After oral consumption, the first positive results were observed after 2 h, and the test remained positive for up to 72 h post-consumption. For I3 it was positive between 1 h and 2 h. These data show that there may be potential problems in recognizing the acute phase, especially in the case of inhalative consumption, which might be problematic especially in traffic controls. The fluctuating results in the case of I2 can be explained by a concentration slightly above the detection limit of the test, resulting from a highly diluted urine at time point 3 h. The creatinine concentration in this sample was only 601 µmol/L and thus distinctly lower than the concentrations of the other samples.

The Immunalysis™ ELISA for serum was also established for the detection of at least 3 ng/mL THC-COOH. With the exception of case I1, the ELISA was positive for at least 24 h post-consumption in all participants. Additionally, a good detection of the acute phase was observed for all cases. For participants O1, O2, O3, and I2 the test was positive up to the last serum sampling point. Therefore, the detectability could be possibly distinctly longer than reported here. As previous spike experiments showed, ELISA testing in serum seems to be beneficial to detect HHC consumption as well as acute phase compared to point-of-care tests^7^. However, obviously ELISA test performances are more time-consuming and require special test equipment which cannot be carried out during police controls.

Overall, it has to be stated that all immunological tests were able to detect single HHC consumption. However, as described above, each test has its own characteristics which has to be taken into account.

### Limitations of the study

The study only included a small number of subjects which limits statistical analyses. With this small study cohort, the influence of inter-individual differences cannot be finally evaluated. The results are therefore to be considered as preliminary. To confirm these previous findings, further studies with a larger number of participants are necessary. Additionally, only low doses and one-time consumption were examined. For higher doses and multiple intakes, significantly higher concentrations, longer detection periods and more severe impairments are conceivable. In addition, in this study only the parent substance and two of the main metabolites were investigated. Future studies with an expanded metabolite spectrum might be useful for further findings.

## Conclusions

In this study, pharmacokinetic data for HHC and its two metabolites 11-OH-HHC and HHC-COOH were investigated in six individuals. Overall, pharmacokinetic characteristics of HHC seem to be similar to THC. As expected, differences between oral and inhalative consumption could be shown. Although there were clear differences between subjects, the psychoactive effects of HHC were evident even at the low doses tested. Therefore, the assumption that HHC consumption bears a potential risk to road safety can be confirmed. Further, similar studies with more participants and a placebo-controlled design are necessary to evaluate these preliminary data.

## Electronic supplementary material

Below is the link to the electronic supplementary material.

Supplementary Material 1

## Data Availability

The datasets generated during and/or analyzed during the current study are available from the corresponding author on reasonable request.
