# Supplementary material for: Preliminary pharmacokinetic and psychophysical investigations after controlled oral and inhalative consumption of hexahydrocannabinol (HHC)
Source: Sci Rep. 2025 Mar 24;15:10086. doi: 10.1038/s41598-025-93931-4 (PMC11933371; doi:10.1038/s41598-025-93931-4)
Supplement: Supplementary file 1 — Supplementary Material 1 [file 41598_2025_93931_MOESM1_ESM.pdf]

Supplementary Information

Preliminary pharmacokinetic and psychophysical investigations after controlled oral and inhalative consumption of hexahydrocannabinol (HHC)

Lisa Höfert[1*], Benjamin Franz[1], Cedric Groß[1], Delen Kuntze[1], Bronislav Jurásek[2], Martin Kuchař[2], Jan Dreßler[1], Susen Becker[#1], and Sven Baumann[#1]

[*] corresponding author (lisa.hoefert@medizin.uni-leipzig.de)
[#] contributed equally

[1] Institute of Forensic Medicine, Medical Faculty, University of Leipzig, Leipzig, Germany
[2] Forensic Laboratory of Biologically Active Substances, Department of Chemistry of Natural Compounds, University of Chemistry and Technology Prague, Prague, Czech Republic

Supplemental Tables

*Table S1: Sample collection schedule for serum, saliva, and urine following inhalative or oral consumption; *=additional conduction of DrugWipe® 5S saliva test*

	**Inhalative**			**Oral**		
	**Serum**	**Saliva**	**Urine**	**Serum**	**Saliva**	**Urine**
	Before consumption*			Before consumption*		
	After first inhalation*			After swallow*		
Minutes	3	3*				
	6	6				
	9	9		10	10*	
	12	12*				
	15	15				
	18	18				
	21	21		20	20	
	24	24				
	27	27				
	30	30*	30	30	30*	
	35	35				
	40	40		40	40	
	45	45				
	50	50		50	50	
	55	55				
Hours	1	1*	1	1	1	1
	1.25	1.25		1.25	1.25	
	1.5	1.5		1.5	1.5*	
	1.75	1.75		1.75	1.75	
	2	2	2	2	2	2
				2.25	2.25	
	2.5	2.5		2.5	2.5	
				2.75	2.75	
	3	3*	3	3	3*	3
	4	4	4	4	4*	4
	5	5		5	5	
	6	6*	6	6	6*	6
	7	7		7	7	
	8	8	8	8	8	8
			10			10
			12			12
	24	24	24	24	24	24
	48	48	48	48	48	48
			72			72
			96			96
			120			120

Table S2: Calibration levels of the analytes in serum

Level, ng/mL	(9*R*)-/(9*S*)-HHC	(9*R*)+(9*S*)-11-OH-HHC	(9*R*)-/(9*S*)-HHC-COOH
1	0.1	-	-
2	0.25	0.25	0.25
3	0.5	0.5	0.5
4	1	1	1
5	2.5	2.5	2.5
6	5	5	5
7	10	10	10
8	25	20	25
9	50	50	50
10	100	-	100

Table S3: Validation data for serum (correlation coefficient r, LOD, LLOQ, QC concentrations, accuracy, intra-day and inter-day precision, recovery, matrix effects; L=low, H=high)

Analyte	r	LOD, ng/mL	LLOQ, ng/mL	QC concen-tration, ng/mL		Accuracy, %		Intra-day precision CV, %		Inter-day precision CV, %		Recovery, %		Matrix effects, %	
				L	H	L	H	L	H	L	H	L	H	L	H
(9*R*)-HHC	0.9998	0.07	0.10	0.80	40.0	95.0	116.4	2.9	1.2	5.6	2.0	41.2	42.9	94.9	77.0
(9*S*)-HHC	0.9996	0.09	0.10	0.80	40.0	113.8	116.6	2.6	1.2	2.9	2.3	44.2	45.0	89.8	75.4
(9*R*)+(9*S*)-11-OH-HHC	0.9969	0.08	0.25	0.80	40.0	95.7	96.1	4.6	1.7	10.6	10.6	79.4	82.5	111.6	99.2
(9*R*)-HHC-COOH	0.9990	0.05	0.25	8.00	80.0	109.6	117.0	2.8	1.7	2.4	2.4	75.9	79.1	99.0	93.9
(9*S*)-HHC-COOH	0.9993	0.08	0.25	8.00	80.0	102.7	113.2	2.0	2.3	4.4	4.2	79.9	79.6	101.8	97.8

Table S4: Validation data for urina and saliva (ranges of calibration, correlation coefficient r, LOD, LLOQ, accuracy, intra-day and inter-day precision; L=low, H=high)

Matrix	Analyte	Range of calibration, ng/mL	r	LOD, ng/mL	LLOQ, ng/mL	QC concentration, ng/mL		Accuracy, %		Intra-day precision CV, %		Inter-day precision CV, %	
						L	H	L	H	L	H	L	H
Urine	(9*R*)-HHC	0.10-25.0	0.9980	0.06	0.10	1.00	10.0	92.9	88.8	1.45	1.85	10.2	8.38
	(9*S*)-HHC	0.10-25.0	0.9963	0.06	0.10	1.00	10.0	95.1	92.3	2.37	2.15	12.9	9.41
	(9*R*)+(9*S*)-11-OH-HHC	0.25-25.0	0.9941	0.25	0.25	1.00	10.0	110.7	102.2	1.82	2.83	12.4	6.31
	(9*R*)-HHC-COOH	0.25-25.0	0.9961	0.07	0.25	1.00	10.0	114.4	117.5	2.99	3.14	8.09	4.84
	(9*S*)-HHC-COOH	0.25-25.0	0.9965	0.11	0.25	1.00	10.0	115.6	114.0	2.91	3.83	6.63	3.72
Saliva	(9*R*)-HHC	2.50-250	0.9996	0.60	2.50	10.0	100	109.5	102.7	3.28	3.28	7.44	9.71
	(9*S*)-HHC	2.50-250	0.9965	0.70	2.50	10.0	100	110.9	102.3	2.48	2.86	7.34	10.5
	(9*R*)+(9*S*)-11-OH-HHC	-	-	2.30	-	-	-	-	-	-	-	-	-
	(9*R*)-HHC-COOH	-	-	0.50	-	-	-	-	-	-	-	-	-
	(9*S*)-HHC-COOH	-	-	0.70	-	-	-	-	-	-	-	-	-

Table S5: Parameters of the scheduled multiple reaction monitoring method

**Analyte**	**Retention time, min**	**Transition, m/z**	**Declustering potential (DP), V**	**Collision energy (CE), V**	**Collision cell exit potential (CXP), V**
(9*R*)-HHC	13.15	317.2 → 193.2	80	35	16
		317.2 → 123.1	80	47	16
(9*S*)-HHC	13.01	317.2 → 193.2	80	35	16
		317.2 → 123.1	80	47	16
11-OH-HHC	8.34	333.2 → 193.2	80	33	12
		333.2 → 123.1	80	35	14
(9*R*)-HHC-COOH	7.95	347.2 → 193.2	80	37	28
		347.2 → 121.2	80	40	10
(9*S*)-HHC-COOH	8.26	347.2 → 193.2	80	37	28
		347.2 → 121.2	80	40	10
Δ9-THC	12.37	315.2 → 193.1	80	31	16
		315.2 → 123.1	80	43	16
Δ9-11-OH-THC	8.02	331.2 → 313.2	80	21	20
		331.2 → 193.1	80	33	13
Δ9-THC-COOH	8.14	345.2 → 327.2	80	23	22
		345.2 → 299.2	100	27	13
CBD	10.19	315.2 → 193.1	80	31	16
		315.2 → 123.1	60	60	12
7-OH-CBD	5.93	331.2 → 313.2	80	17	8
		331.2 → 105.1	80	51	12
7-COOH-CBD	5.66	345.3 → 327.2	80	21	24
		345.3 → 299.2	80	27	8
CBN	11.59	311.2 → 223.1	80	29	16
		311.2 → 293.2	80	25	13
(9*R*)-HHC-D9	13.15	326.3 → 202.2	80	35	16
		326.3 → 123.0	80	37	16
Δ9-THC-D3	12.37	318.2 → 196.1	80	31	16
		318.2 → 123.1	80	43	16
Δ9-11-OH-THC-D3	8.02	334.2 → 316.2	80	21	20
		334.2 → 196.1	80	33	13
Δ9-THC-COOH-D3	8.14	348.2 → 330.2	80	23	22
		348.2 → 302.2	100	27	13

Supplemental Figures

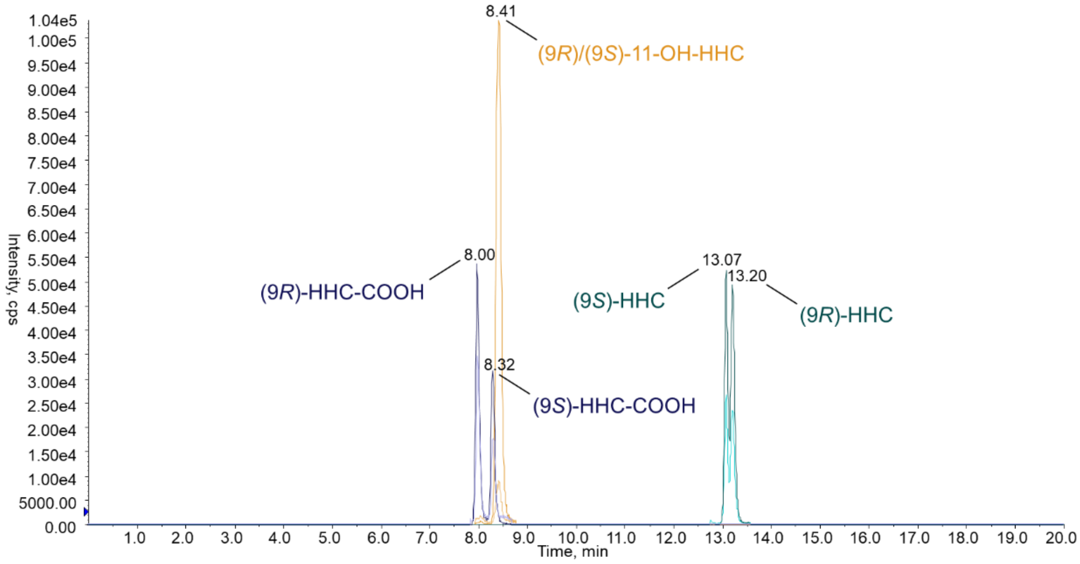

Figure S1: Extracted ion chromatogram for a serum sample spiked with 2.5 ng/mL for each analyte (calibrator 5)

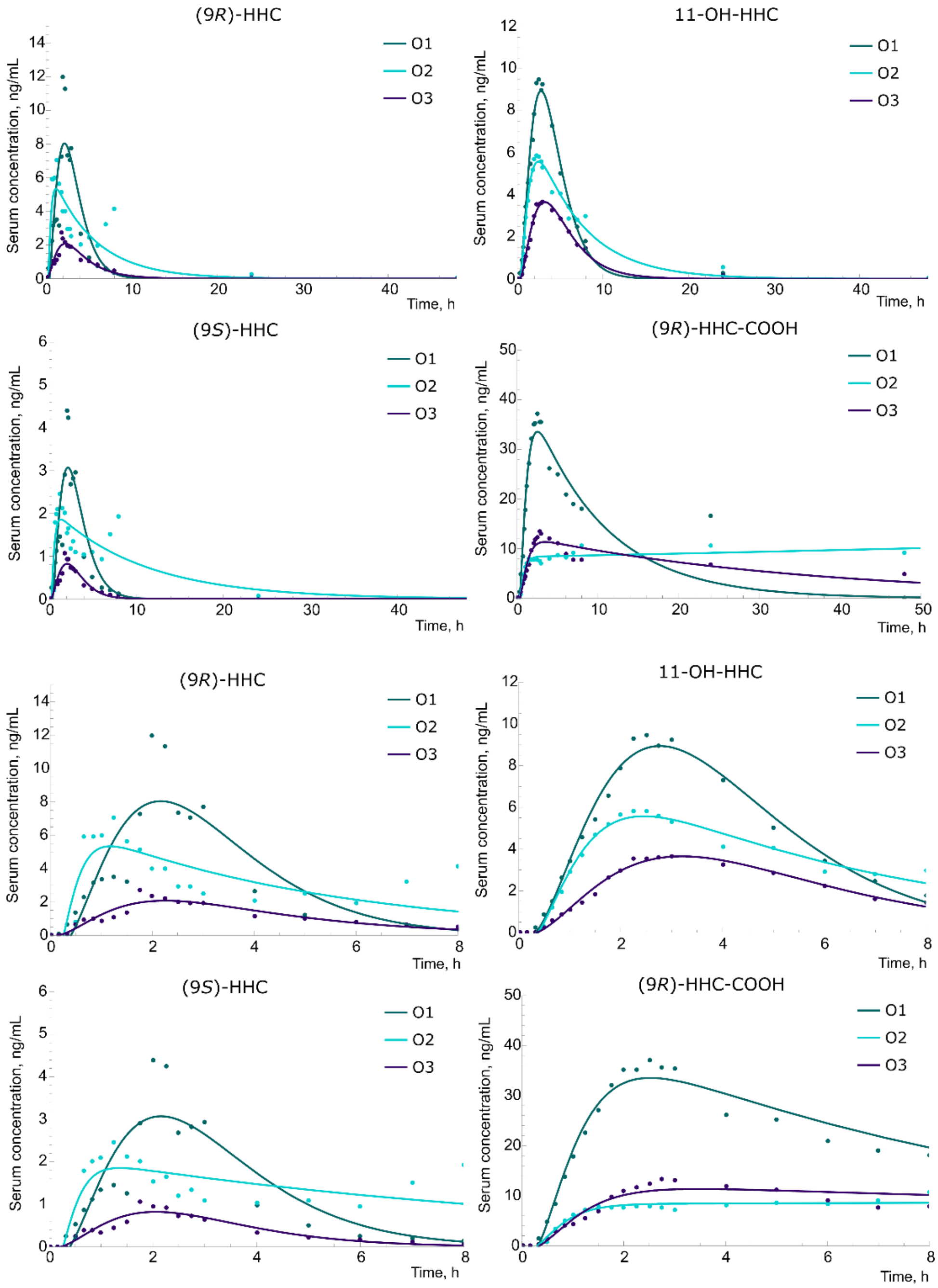

Figure S2: Compartmental analysis for the oral consumption group for (9R)-HHC, (9S)-HHC, 11-OH-HHC, and (9R)-HHC-COOH in serum using the two-compartmental model with first-order absorption

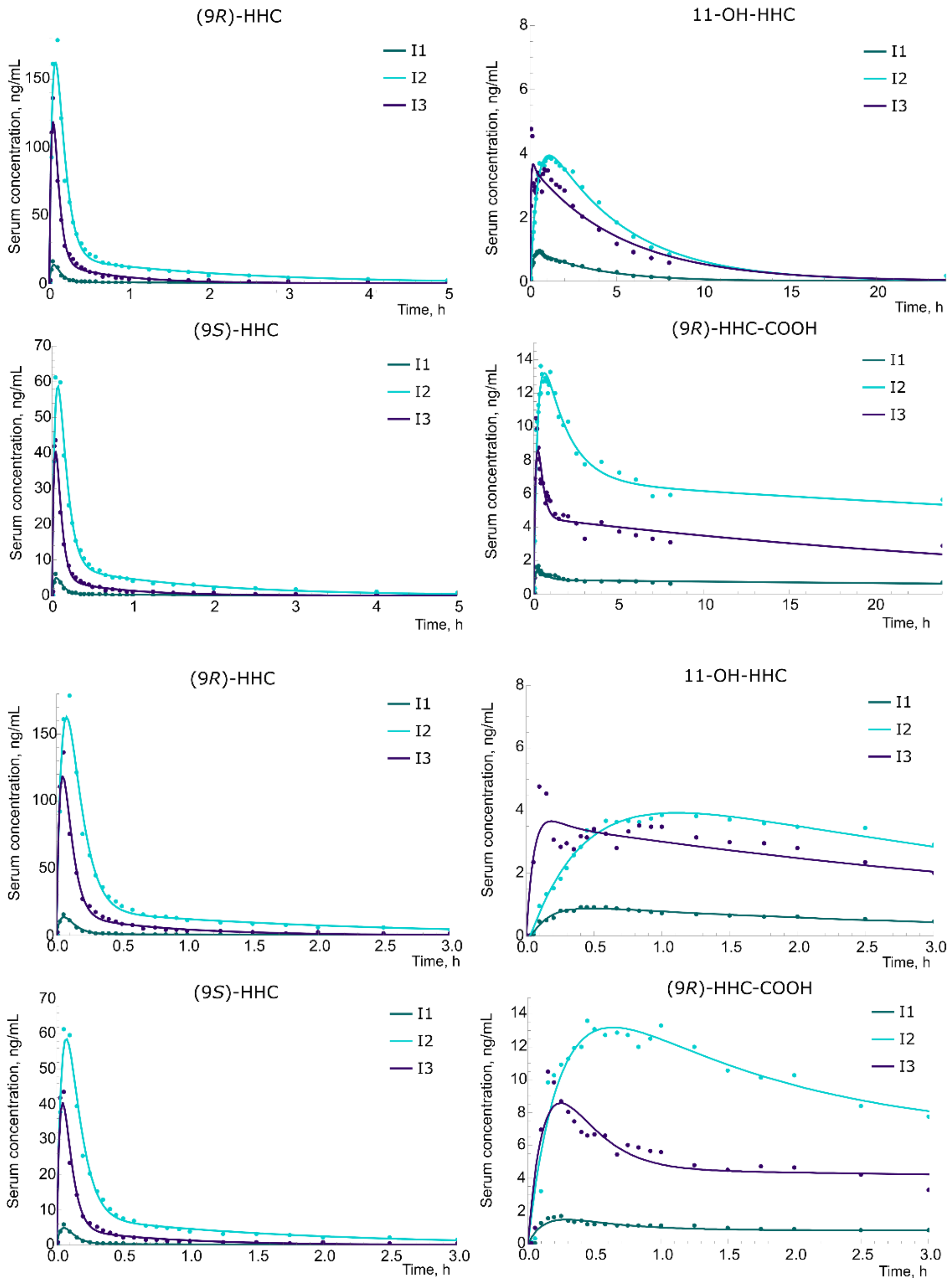

Figure S3: Compartmental analysis for the inhalative consumption group for (9R)-HHC, (9S)-HHC, 11-OH-HHC, and (9R)-HHC-COOH in serum using the two-compartmental model with first-order absorption
